# Anal incontinence after a prolonged second stage of labor in primiparous women

**DOI:** 10.1038/s41598-022-11346-x

**Published:** 2022-05-05

**Authors:** Sandra Bergendahl, Anna Sandström, Alexandra Spasojevic, Sophia Brismar Wendel

**Affiliations:** 1 Department of Clinical Sciences, Karolinska Institutet, Danderyd Hospital, Stockholm, Sweden; 2 Department of Women’s Health, Danderyd Hospital, 18288 Stockholm, Sweden; 3 Clinical Epidemiology Division, Department of Medicine, Karolinska Institutet, Stockholm, Sweden; 4 Department of Women’s Health, Karolinska University Hospital, Stockholm, Sweden

**Keywords:** Urogenital reproductive disorders, Faecal incontinence, Epidemiology

## Abstract

The objective was to investigate the effect of delivery mode on anal incontinence 1–2 years after delivery in primiparous women with prolonged second stage of labor. This population-based cohort and questionnaire study performed in Stockholm Region, Sweden, included 1302 primiparous women with a second stage ≥ 3 h from December 1st, 2017 through November 30th, 2018. Background characteristics and outcome data were retrieved from computerized records. Questionnaires based on Wexner score were distributed 1–2 years after delivery. Risk of anal incontinence, defined as Wexner score ≥ 2, was calculated using logistic regression and presented as crude and adjusted odds ratios (OR and aOR) with 95% confidence intervals (CI). Compared with cesarean section, vacuum extraction was associated with anal incontinence (aOR 2.25, 95% CI 1.21–4.18) while spontaneous delivery was not (aOR 1.55, 95% CI 0.85–2.84). Anal incontinence was independently associated with obstetric anal sphincter injuries (aOR 2.03, 95% CI 1.17–3.5) and 2nd degree perineal tears (aOR 1.36, 95% CI 1.03–1.81) compared with no or 1st degree perineal tear. Obstetric anal sphincter injury at vacuum extraction inferred the highest risk of anal incontinence (aOR 4.06, 95% CI 1.80–9.14), compared with cesarean section. Increasing duration of the prolonged second stage did not affect the risk.

## Introduction

Second stage of labor in nulliparous women is defined as prolonged when exceeding 3 h^1–5^, and occurs in 10–20% of nulliparous women^1,6,7^. With contemporary labor curves and clinical management, less than 10% of nulliparous women have a second stage exceeding 4 h^8,9^.

A prolonged second stage has been associated with obstetric anal sphincter injuries (OASIS)^1,6,7,10–12^, postpartum hemorrhage^1,6,10,12–14^, maternal infection^1,6,10,12,15^, operative vaginal delivery^1,16,17^, and cesarean section^1,16,17^. A prolonged second stage has also been associated with low Apgar score^18,19^, neonatal sepsis^12,18^, and admission to neonatal intensive care^12,18^. These complications may be directly or indirectly related to interventions in the second stage, and it has been suggested to wait rather than to vacuum^20^. However, a severe complication after very prolonged, obstructed labor is the formation of vesicovaginal and rectovaginal fistulas^21^. Thus, a remaining clinical dilemma is that no upper time limit has been established. The American College of Obstetricians and Gynecologists states that the duration could be extended as long as progress is documented^22,23^. Neither the WHO, nor the National Institute for Health and Care Excellence present an upper time limit^4,24^.

Adding to the clinical dilemma, the recommended mode of delivery in a prolonged second stage has not been defined^23,25,26^. A prolonged second stage, operative vaginal delivery, and OASIS are often sequential^7^, and OASIS is known to cause anal incontinence^27,28^. It has been suggested that a prolonged second stage in itself has long-term effects on the pelvic floor by compressing the pudendal nerve or irreversibly widening the levator hiatus^29^. It has also been suggested that operative vaginal delivery in itself increases the risk of anal incontinence^30^.

While a second stage of 3 h is acceptable and should not motivate intervention, there is lack of knowledge regarding when to intervene after this duration, and how to best expedite delivery to avoid pelvic floor dysfunction. The aim of this study was to investigate the effect of mode of delivery on patient-reported anal incontinence 1–2 years after a first delivery with a second stage of labor exceeding 3 h.

## Material and methods

### Study population and exposure

The source population was all women who gave birth in any of the six labor ward hospitals in Stockholm, Sweden, from December 1st, 2017 through November 30th, 2018. The deliveries in Stockholm constitute approximately 25% of all deliveries in Sweden^31^. All hospitals have similar facilities and staffing and use the same computerized medical records system (Obstetrix, Cerner Corporation, Sweden) with the same evaluation standards supported by documentation templates and compulsory checkboxes. Virtually all deliveries take place in the hospitals and are attended by midwives, with residents and consultants available at all times. All hospitals have operating facilities that are immediately available. All obstetric care is tax funded and free of charge. The medical records cover antenatal care, labor and delivery, and postnatal care for all births in Stockholm. At the time of data collection there was neither a regional nor national guidline regarding management of the second stage. The common practice in Sweden is to await the woman’s spontaneous urge to push. Oxytocin is administered after 1–2 h without progress in the passive phase of second stage or after 30 min of inefficient pushing. Active pushing is encouraged after 3 h without progress in the passive phase.

Inclusion criteria in the study were primiparous women with a term (≥ 37 gestational weeks), live, single, cephalic birth, and a prolonged second stage of labor ≥ 3 h. Maternal and neonatal characteristics and outcomes were retrieved from computerized medical records by the Stockholm Region Enterprise Data Warehouse. We chose a 3-h duration cut-off since a shorter duration is considered normal^1–4,8,32^, and thereby the duration in itself is not considered an indication for intervention before 3 h.

**Figure 1:**
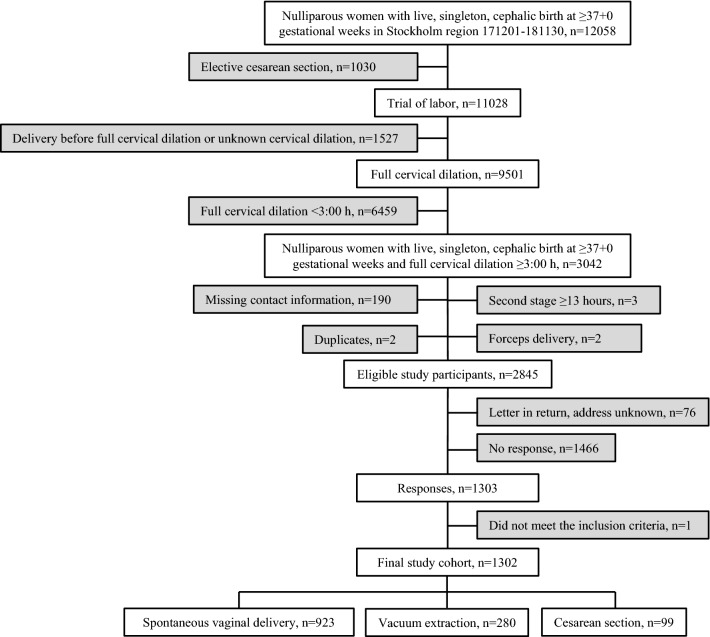
Flowchart.

During the stated time-period there were 12,058 primiparous women with a term, live, single, cephalic birth. We excluded women with an elective cesarean section (n = 1030). Second stage of labor duration was calculated from two time points in the partograph: time of birth and the timepoint of first notation of full cervical dilation. We excluded women who had unknown cervical dilation and those with full cervical dilation for < 3 h (Fig. 1). The remaining 3042 women met the inclusion criteria. For these women, we retrieved contact information, information on delivery mode, and second stage duration. Women with missing contact information (n = 190), duplicate registrations (n = 2), forceps delivery (n = 2), and outliers with a second stage exceeding 13 h (n = 3) were not further analyzed (Fig. 1).

In total, 2845 women were eligible study participants (Fig. 1). They were sent a letter containing study information, a consent form, and a questionnaire on pelvic floor function^33^. One postal reminder was sent after 2 weeks. Letters were sent and received from December 1st, 2019 through June 30th, 2020 ensuring a time interval of 1–2 years after delivery.

Main exposure was mode of delivery after a prolonged second stage (≥ 3 h): spontaneous vaginal delivery, vacuum extraction, and cesarean section. Secondary exposures were duration of second stage, duration of fetal head station below the ischial spines, and perineal injury.

### Outcome measures

The questionnaire used in the study is based on the 1-year follow-up questionnaire developed and used in the Swedish Perineal Tear Register, containing questions about urinary incontinence, anal incontinence, symptoms of pelvic organ prolapse, and sexual function^33^. Anal incontinence is measured by Wexner score^34^, containing the following parameters: incontinence of flatus, liquid or solid stool, the use of pads, and alteration of lifestyle. Each parameter is assessed with a scoring system (never = 0, rarely = 1, sometimes = 2, usually = 3, and always = 4). Maximum score is 20^34^. Wexner score is the most cited anal incontinence score^35^. It was developed as an interview-based score but has been validated also as a self-reporting scoring system with highly consistent results compared to the results obtained by interview^36^. Jangö et al.^37^ evaluated Wexner score in women with previous OASIS and found Wexner score ≥ 2 to be a significant cut-off for affected quality of life. Thus, we chose Wexner score ≥ 2 as our primary outcome. Women with a subsequent delivery were instructed to answer the questions based on symptoms before the second pregnancy.

### Covariates

Maternal age (< / ≥ 35 years), height (< / ≥ 155 cm), body mass index (BMI; </≥ 30 kg/m^2^), country of birth (Sweden/other European country/country outside Europe), cohabitation (yes/no), tobacco use in early pregnancy (yes/no), and intercurrent diseases (diabetes, morbus Crohn/ulcerative colitis or asthma; yes/no) were registered by the midwife at the first antenatal visit in gestational week 8–12. Gestational age at birth was determined by a second trimester ultrasound and categorized in 2-week intervals (37–38, 39–40, 41–42 weeks).

Delivery characteristics were recorded by the attending midwife or physician: mode of onset (spontaneous start or induction of labor), epidural anesthesia (yes/no), time point of full cervical dilation, fetal station in relation to the ischial spines, time of birth, and oxytocin augmentation (yes/no). The second stage duration was categorized into 1-h intervals (3:00–3:59, 4:00–4:59, 5:00–5:59 and ≥ 6:00 h and minutes). Duration of fetal station below the ischial spines was calculated from the first registration of any station below the ischial spines until birth. Lack of registration of fetal station below the ischial spines could either be because the fetus did not reach this station or because no vaginal examination was registered. It was in both cases included and categorized as “unknown”. Duration below the ischial spines was categorized into 1-h intervals (0:00–0:59, 1:00–1:59, 2:00–2:59, 3:00–3:59. 4:00–4:59, ≥ 5:00 h and minutes). Delivery outcomes were recorded by the attending midwife and categorized: Birthweight (</≥ 4000 g), head circumference (</≥ 38 cm), and fetal position (occiput anterior/occiput posterior/other).

Episiotomy, perineal and vaginal tears were classified according to the International Classification of Diseases-10 (ICD-10) Swedish Edition by clincical examination. We collected information on degree of injury from diagnosis codes and procedure codes. We categorized women into three groups according to their most severe injury: 1st degree perineal tear (O70.0), including women with no diagnosis and women with an isolated vaginal tear (O71.4), 2nd degree perineal tear (O70.1) also including women with only an episiotomy diagnosis (TMA00), and OASIS including 3rd (O70.2) and 4th degree perineal tears (O70.3).

### Statistical analyses

Statistical analyses were performed using SPSS 27.0 (IBM, Armonk, NY, USA).

Maternal characteristics, delivery characteristics, and outcomes in women with Wexner score ≥ 2 compared with < 2 were calculated and compared by Chi^2^ test. Median Wexner score was calculated by Kruskal–Wallis test. A *p* value < 0.05 was considered significant. Relative risk of Wexner score ≥ 2 compared with < 2 was calculated using complete case analysis with unconditional univariate and multivariate logistic regression with cesarean section as the reference for the main exposure ‘mode of delivery’. Results are presented as crude and adjusted odds ratios (OR and aOR) with 95% confidence intervals (CI). We used directed acyclic graphs (DAG) to select possible covariates for the adjusted analyses. Different DAGs were created for the main exposure ‘mode of delivery’ and for the secondary exposures, ‘duration of second stage/duration of fetal station below the ischial spines’, ‘perineal injury’, and the outcome Wexner score ≥ 2 (Supplementary Fig. S1). We included two different models for adjustment. In model A, adjustments were made for the potential confounders, but not the mediator pathway. In model B adjustements were made for the potential confounders and for the mediator pathway (Supplementary Fig. S1). The potential interaction between delivery mode and perineal injury on Wexner score ≥ 2 was calculated and presented as prevalence, proportions, and unadjusted odds ratios using univariate logistic regression with the terms ‘delivery mode’, ‘perineal injury’ and the interaction term ‘delivery mode*perineal injury’. Cesarean section was used as reference.

Missing data is presented in the tables when prevalent. Missing data on duration of fetal station below the ischial spines was categorized as “unknown” and missing data on perineal or vaginal tears was categorized as “no diagnosis”, to avoid exclusion in regression analyses. Missing data in other variables constituted a minor proportion (< 3%) except for BMI (4.2%). We performed sensitivity analyses with multiple imputation of the covariate BMI with no difference compared with the presented results (data not presented). We also performed sensitivity analyses excluding women with a subsequent delivery during the follow-up period, and women who answered the questionnaire later than 24 months after delivery. No difference was found compared with the presented results (data not presented). Therefore all women were included.

### Ethical considerations

The study was approved by the Regional Ethical Review Board in Stockholm on 17 June 2015 (2015/887‐31/4) with amendments 20 August 2018 (2018/1538-32), 2 May 2019 (2019-01587), and 3 February 2020 (2019-01587). In accordance with the General Data Protection Regulation, we obtained written consent from the Head of Department in all participating hospitals (Danderyd Hospital, South General Hospital, Karolinska University Hospital Huddinge and Solna, Sodertalje Hospital, and BB Stockholm) in order to retrieve data from medical records. This study is reported according to the Strengthening the Reporting of Observational Studies in Epidemiology (STROBE) guideline^38^.

## Results

We received 1302 responses of 2845 distributed questionnaires (response rate 45.8%), while 76 questionnaires were returned to sender due to unknown address. One woman had been included despite a breech presentation and was excluded before analyses (Fig. 1). The final study cohort consisted of 1302 primiparous women with a live, single cephalic birth at ≥ 37 gestational weeks and a second stage duration ≥ 3 h. Of these, 923 (70.9%) women had a spontaneous vaginal delivery, 280 (21.5%) a vacuum extraction, and 99 (7.6%) a cesarean section (Fig. 1). OASIS was diagnosed in 37 (4.0%) women with spontaneous vaginal delivery and in 35 (12.5%) women with vacuum extraction. This is similar to the national average for primiparous women^31^.

**Figure 2:**
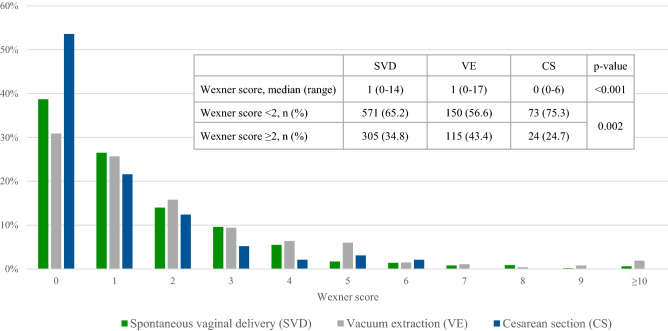
Wexner score and mode of delivery.

Women with Wexner score ≥ 2 were older than women with Wexner score < 2, but otherwise similar (Table 1). The prevalence of Wexner score ≥ 2 was highest after vacuum extraction, followed by spontaneous vaginal delivery, and lowest after cesarean section (Fig. 2). Wexner score medians and ranges were significantly higher in both vaginal modes of delivery compared with cesarean section (Fig. 2). Characteristics and outcomes according to mode of delivery can be found as Supplementary Table S1 and S2.

**Table 1:** Characteristics of women with Wexner score < 2 and ≥ 2.

	Wexner score < 2 n (%) 794 (64.1)	Wexner score ≥ 2 n (%) 444 (35.9)	*p* value
**Age (years)**			
< 35	652 (66.1)	335 (33.9)	0.005
≥ 35	142 (56.6)	109 (43.4)	
**Height (cm)**			
< 155	15 (71.4)	6 (28.6)	0.484
≥ 155	766 (64.0)	430 (36.0)	
Missing	13 (61.9)	8 (38.1)	
**BMI (kg/m**^**2**^**)**			
< 30	719 (64.8)	391 (35.2)	0.285
≥ 30	44 (58.7)	31 (41.3)	
Missing	31 (58.5)	22 (41.5)	
**Country of birth**			
Sweden	754 (64.1)	422 (35.9)	0.941
Other European country	20 (62.5)	12 (37.5)	
Outside Europe	20 (66.7)	10 (33.3)	
**Cohabitation**			
Yes	738 (64.5)	407 (35.5)	0.652
No	45 (59.2)	31 (40.8)	
Missing	6 (35.3)	11 (64.7)	
**Tobacco use in early pregnancy**			
Yes	9 (75.0)	3 (25.0)	0.652
No	754 (63.9)	426 (36.1)	
Missing	31 (67.4)	15 (32.6)	
**Diabetes**			
Yes	6 (75.0)	2 (25.0)	0.454
No	768 (64.3)	426 (35.7)	
Missing	20 (55.6)	16 (44.4)	
**Morbus Crohn/Ulcerative colitis**			
Yes	4 (57.1)	3 (42.9)	0.510
No	770 (64.4)	425 (35.6)	
Missing	20 (55.6)	16 (44.4)	
**Asthma**			
Yes	53 (60.2)	35 (39.8)	0.386
No	721 (64.7)	393 (35.3)	
Missing	20 (55.6)	16 (44.4)	
**Time since delivery (months)**			
< 18	72 (61.5)	45 (38.5)	0.533
≥ 18	716 (64.4)	395 (35.6)	
Missing	6 (60.0)	4 (40.0)	
**Subsequent delivery**			
No	684 (63.7)	389 (36.3)	0.636
Yes	96 (65.8)	50 (34.2)	
Missing	14 (73.7)	5 (26.3)	

64 women (4.9%) had missing data on Wexner score.

Missing values presented when prevalent. *P* values are calculated using Chi^2^ tests excluding missing values.

In women with vaginal delivery, the prevalence of Wexner score ≥ 2 successively increased with degree of perineal injury (Table 2). Women with episiotomy only had the lowest prevalence of Wexner score ≥ 2 (Table 2). Neither second stage duration, duration of fetal station below the ischial spines, fetal position, birthweight, nor head circumference affected the prevalence of Wexner score ≥ 2 (Table 2). When collapsing durations over 5 h into one group, no significant association was seen (data not presented).

**Table 2:** Delivery characteristics and outcomes in women with Wexner score < 2 and ≥ 2.

	Wexner score < 2 n (%) 794 (64.1)	Wexner score ≥ 2 n (%) 444 (35.9)	*p* value
**Gestational age (weeks)**			
37–38	115 (68.9)	52 (31.1)	0.365
39–40	420 (63.0)	247 (37.0)	
41–42	259 (64.1)	145 (35.9)	
**Mode of onset**			
Induction of labor	191 (61.2)	121 (38.8)	0.214
Spontaneous onset	603 (65.1)	323 (34.9)	
**Epidural**			
Yes	657 (65.0)	353 (35.0)	0.158
No	137 (60.1)	91 (39.9)	
**Duration of second stage (h:mm)**			
3:00–3:59	408 (65.3)	217 (34.7)	0.343
4:00–4:59	251 (63.4)	145 (36.6)	
5:00–5:59	92 (59.0)	64 (41.0)	
≥ 6:00	43 (70.5)	18 (29.5)	
**Oxytocin augmentation**			
Yes	749 (64.2)	417 (35.8)	0.766
No	45 (62.5)	27 (37.5)	
**Duration of fetal station below the ischial spines (h:mm)**			
0:00–00:59	124 (59.3)	85 (40.7)	0.135
1:00–1:59	139 (59.9)	93 (40.1)	
2:00–2:59	102 (61.8)	63 (38.2)	
3:00–3:59	174 (69.9)	75 (30.1)	
4:00–4:59	84 (65.6)	44 (34.4)	
≥ 5:00	55 (63.2)	32 (36.8)	
‘Unknown’	116 (69.0)	52 (31.0)	
**Mode of delivery**			
Cesarean section	73 (75.3)	24 (24.7)	0.002
Spontaneous delivery	571 (65.2)	305 (34.8)	
Vacuum extraction	150 (56.6)	115 (43.4)	
**Birthweight (g)**			
< 4000	639 (64.2)	356 (35.8)	0.865
≥ 4000	154 (63.6)	88 (36.4)	
Missing	1 (100)	0	
**Head circumference (cm)**			
< 38	747 (63.9)	422 (36.1)	0.255
≥ 38	42 (71.2)	17 (28.8)	
Missing	5 (50.0)	5 (50.0)	
**Fetal position**			
Occiput anterior	711 (63.3)	412 (36.7)	0.232
Occiput posterior	55 (69.6)	24 (30.4)	
Other	17 (77.3)	5 (22.7)	
Missing	11 (78.6)	3 (21.4)	
**Perineal injury**^**a**^			
Degree 0–1^b^	272 (69.4)	120 (30.6)	< 0.001
Degree 2^c^	413 (60.9)	265 (39.1)	
2nd degree only	378 (59.9)	253 (40.1)	
Episiotomy only	35 (74.5)	12 (25.5)	
OASIS	36 (50.7)	35 (49.3)	
3rd degree	34 (50.0)	34 (50.0)	
4th degree	2 (66.7)	1 (33.3)	
**Episiotomy**^**a**^			
Yes	78 (65.0)	42 (35.0)	0.054
No	643 (63.0)	378 (37.0)	

64 women (4.9%) had missing data on Wexner score.

^a^Vaginal deliveries only, Wexner score < 2 n = 721 Wexner score ≥ 2 n = 420.

^b^No diagnosis, 1st degree or isolated vaginal tear.

^c^2nd degree including episiotomy. Missing values presented when prevalent. *P* values are calculated using Chi^2^ tests excluding missing values.

Vacuum extraction was associated with Wexner score ≥ 2, also after adjusting for perineal injury (Table 3). Spontaneous vaginal delivery was not associated with a Wexner score ≥ 2 after adjustments (Table 3). The risk of Wexner score ≥ 2 was increased in women with OASIS and women with a 2nd degree tear or episiotomy compared with women with no or 1st degree perineal injury (Table 3).

**Table 3:** Univariate and multivariate logistic regression for Wexner score ≥ 2.

	OR (95% CI)	aOR (95% CI) (Model A)	aOR (95% CI) (Model B)
**Mode of delivery**			
Cesarean section	1.0 (ref)	1.0 (ref)	1.0 (ref)
Spontaneous delivery	1.63 (1.00–2.63)	1.55 (0.85–2.84)^a^	1.47 (0.80–2.69)^b^
Vacuum extraction	2.33 (1.39–3.93)	2.25 (1.21–4.18)^a^	1.96 (1.03–3.73)^b^
**Duration of second stage (h:mm)**			
3:00–3:59	1.0 (ref)	1.0 (ref)	1.0 (ref)
4:00–4:59	1.09 (0.84–1.41)	1.07 (0.81–1.41)^c^	1.01 (0.77–1.34)^d^
5:00–5:59	1.31 (0.91–1.87)	1.35 (0.93–1.97)^c^	1.38 (0.94–2.03)^d^
≥ 6:00	0.79 (0.44–1.40)	0.69 (0.38–1.28)^c^	0.75 (0.40–1.42)^d^
**Duration below the ischial spines (h:mm)**			
0:00–00:59	1.0 (ref)	1.0 (ref)	1.0 (ref)
1:00–1:59	0.98 (0.67–1.43)	0.98 (0.66–1.46)^c^	0.97 (0.65–1.44)^d^
2:00–2:59	0.90 (0.59–1.37)	0.90 (0.58–1.39)^c^	0.88 (0.57–1.37)^d^
3:00–3:59	0.63 (0.43–0.93)	0.64 (0.43–0.96)^c^	0.65 (0.43–0.97)^d^
4:00–4:59	0.76 (0.48–1.20)	0.76 (0.47–1.23)^c^	0.72 (0.44–1.17)^d^
≥ 5:00	0.85 (0.51–1.42)	0.91 (0.53–1.55)^c^	0.89 (0.51–1.53)^d^
‘Unknown’	0.65 (0.43–1.00)	0.70 (0.45–1.09)^c^	0.73 (0.46–1.17)^d^
**Perineal injury**^**e**^			
Degree 0–1^f^	1.0 (ref)	1.0 (ref)	
Degree 2^g^	1.45 (1.12–1.90)	1.36 (1.03–1.81)^h^	
OASIS	2.20 (1.32–3.68)	2.03 (1.17–3.52)^h^	
**Episiotomy**^**e**^			
No	1.0 (ref)	1.0 (ref)	
Yes	0.99 (0.95–1.03)	0.91 (0.59–1.39)^h^	

Unadjusted model: n = 1238 women.

Model A: Confounders adjusted for according to the Model A DAG:s for the different exposures.

Model B: Confounders and mediators adjusted for according to the Model B DAG:s for the different exposures.

^a^Adjusted for age, BMI, Mb Crohn/Ulcerative colitis, second stage duration, duration of fetal station below the ischial spines, fetal position, birthweight, and head circumference (Supplementary Fig. S1 DAG 1 model A), n = 1132.

^b^Adjusted for age, BMI, second stage duration, duration of fetal station below the ischial spines, perineal injury, birthweight, and head circumference (Supplementary Fig. S1 DAG 1 model B), n = 1175.

^c^Adjusted for age, BMI, birthweight, and head circumference (Supplementary Fig. S1 DAG 2 model A), n = 1175.

^d^Adjusted for age, BMI, mode of delivery, perineal injury, birthweight, and head circumference (Supplementary Fig. S1 DAG 2 model B), n = 1175.

^e^Vaginal deliveries only, n = 1204.

^f^No diagnosis, 1st degree or isolated vaginal tear.

^g^2nd degree including episiotomy.

^h^Adjusted for age, BMI, mode of delivery, second stage duration, duration of fetal station below the ischial spines, fetal position, birthweight, and head circumference (Supplementary Fig. S1 DAG 3), n = 1075.

There was an interaction between mode of delivery and perineal injury on the risk of Wexner score ≥ 2 (Table 4). In women with no or 1st degree perineal injury, the risk of Wexner score ≥ 2 was not increased compared with cesarean section. In women with 2nd degree injury or episiotomy the risk of Wexner score ≥ 2 was increased after both modes of vaginal delivery (Table 4). Women with an OASIS at vacuum extraction had a fourfold risk of Wexner score ≥ 2, while this risk increase did not reach statistical significance in women with OASIS in spontaneous vaginal delivery (Table 4).

**Table 4:** Interaction between delivery mode and perineal injury on the risk of Wexner score ≥ 2.

Mode of delivery and Perineal injury	Wexner score < 2 n (%) 721 (63.2)	Wexner score ≥ 2 n (%) 420 (36.8)	OR (95% CI)	*p* value^c^
Cesarean section	73 (75.3)	24 (24.7)	1.0 (ref)	
Spontaneous vaginal delivery and Degree 0–1^a^	236 (69.6)	103 (30.4)	1.33 (0.79–2.22)	0.282
Spontaneous vaginal delivery and Degree 2^b^	314 (62.7)	187 (37.3)	1.81 (1.10–2.97)	0.019
Spontaneous vaginal delivery and OASIS	21 (58.3)	15 (41.7)	2.17 (0.97–4.87)	0.060
Vacuum extraction and Degree 0–1^a^	36 (67.9)	17 (32.1)	1.44 (0.69–3.01)	0.340
Vacuum extraction and Degree 2^b^	99 (55.9)	78 (44.1)	2.40 (1.39–4.15)	0.002
Vacuum extraction and OASIS	15 (42.9)	20 (57.1)	4.06 (1.80–9.14)	0.001

^a^No diagnosis, 1st degree or isolated vaginal tear.

^b^2nd degree including episiotomy.

^c^*P* values according to interaction analysis including the terms ‘delivery mode’, ‘perineal injury’, and ‘delivery mode*perineal injury’.

In total, 147 (11.3%) women had a subsequent delivery during the follow-up period. In the sensitivity analyses, there was no difference in prevalence of Wexner score ≥ 2 between women without a subsequent delivery and the total study cohort (data not presented). Of all 1302 answers, 69 women (5.3%) answered the questionnaire later (25–27 months) than the stipulated 12–24 months after delivery. There was no difference in prevalence of Wexner score ≥ 2 between women with a time since delivery 12–24 months and the total study cohort (data not presented).

## Discussion

### Main findings

This study showed that vacuum extraction but not spontaneous vaginal delivery, compared with cesarean section, increased the risk of anal incontinence 1–2 years after a first delivery with a prolonged second stage, irrespective of degree of perineal injury. We could not show that the duration of prolonged second stage, nor the duration of fetal station below the ischial spines, affected the risk of anal incontinence. In women with vaginal delivery, a perineal injury especially OASIS, was the most important risk factor for anal incontinence. The combination of vacuum extraction and OASIS was the most detrimental to anal continence, without any relation to increasing duration of the prolonged second stage.

### Interpretation

Few other studies have had a comparable approach, differing in exposure or outcome, or are associated with a considerable power problem^29,39,40^. When comparing cesarean section in the second stage to spontaneous vaginal delivery, no difference in long-term anal incontinence has been found^41^. When comparing operative vaginal delivery to spontaneous vaginal delivery after a prolonged second stage, long-term anal incontinence has been found to be similar or increased, especially by OASIS, but not a prolonged second stage^30,42^. Thus, most existing observations support our finding that second stage duration in itself does not affect the risk of anal incontinence, but mode of delivery and its feared consequences, especially OASIS, do.

Apart from when to end a prolonged second stage, the duration of second stage is dependent on the three classic “Ps of labor”: “the pelvis, passenger, and power”^43^. Power includes the modifiable factors contractions and maternal pushing. The use of oxytocin and immediate pushing will significantly shorten the second stage^44,45^. The timing of pushing does however not influence the risk of OASIS^44,45^, or fecal incontinence^46^.

### Clinical implications and further research

Several factors influence the decision on when and how to intervene. The station of the fetal head is essential for the choice between instrumental vaginal delivery or cesarean section, but also assessement of maternal and fetal risk factors for unsuccessful instrumental vaginal delivery followed by a potentially difficult cesarean section. Risk of OASIS, shoulder dystocia, anesthesiological, or surgical complications also influence the desicion. To this, the contractions, maternal pushing efforts, signs of fetal distress, parents’ wish, and the physician’s preferred mode of delivery can be added. Thus the best way forward in a prolonged second stage is a complex jigsaw, to which the results of our study may provide one piece to inform management: The wait and see approach does not in itself increase the risk of anal incontinence.

Our results indicate that the forced passage by operative vaginal delivery through the pelvic floor is the critical event. If a spontaneous vaginal delivery seems possible, non-instrumental management and care to avoid OASIS should be the recommendation. If vacuum extraction is chosen, maximum efforts should be made to avoid OASIS. With regard to protective measures against OASIS which is known to be the most important risk factor of anal incontinence in women^27,28^, it is noteworthy that women with episiotomy had no increased risk of anal incontinence. This supports liberal or even routine use of episiotomy in vacuum extraction in nulliparous women, since a lateral or mediolateral episiotomy in this specified population may half the risk of OASIS^47^. Whether routine use of episiotomy in vacuum extraction in nulliparous women will decrease long-term anal continence remains to be assessed in an ongoing randomized controlled trial^48^.

It has been suggested that levator ani muscle injury contributes to anal incontinence^49^. Prolonged second stage, operative vaginal delivery, and OASIS are associated with levator ani muscle injury^50,51^. At the time of data collection, there was no separate ICD-10 diagnosis code for levator ani muscle injury, and such occult injuries could be an unmeasured confounder. Since January 1st 2020 the Swedish ICD-10 has been updated with specific codes for levator ani muscle injuries which enables further research on the association between these injuries and anal incontinence.

When spontaneous vaginal delivery is no longer an option and vacuum extraction seems unfavorable, cesarean section could be the preferable mode of delivery to prevent future anal incontinence. To test if cesarean section is preferable to vacuum extraction in a prolonged second stage to prevent future anal incontinence, a randomized controlled trial could be conducted. However, it could be a challenge to complete such a trial.

### Strengths and limitations

To our knowledge, this is the largest study investigating the long-term risk of anal incontinence depending on mode of delivery after a prolonged second stage of labor. Our method of extracting data from computerized medical records enabled a large set of variables and duration details which are rarely possible using register data. We used the validated Wexner score to quantify anal incontinence and applied a cut-off developed for a similar population^37^. The time-point of measurement, 12–24 months after delivery, represents a time-point after recovery from childbirth which should reflect symptoms that are likely to remain^52^.

The major limitation was the moderate response rate, entailing a possible self-selection bias. Women with symptoms could be more likely to respond, causing an overestimate of the overall prevalence of anal incontinence. We assessed available data on non-respondents and found no difference compared to respondents in second stage duration or proportion of vacuum extractions, but a slightly smaller proportion of spontaneous vaginal deliveries and larger proportion of cesarean sections. Another important limitation is the risk of lack of power. Some observed associations were not statistically significant, specifically between Wexner score ≥ 2 and an increasing second stage duration, with a decreasing number of women in each duration interval. Albeit, there was no difference if durations longer than 5 h were collapsed into one interval. Thus, we cannot exclude an association between anal incontinence and very long second stage durations. That said, we considered 1-year data to be sufficient to explore if there were clinically important differences.

Another limitation was that the questionnaire was only available in Swedish. This could result in an availability bias due to language barrier. This was reflected in a somewhat lower response rate in women delivered in hospitals with a higher proportion of women with another primary language. Also, one limitation was that other variables that potentially could affect the results such as maternal exercise habits, education, or occupation was not available or poorly reported in the data source and hence could not be studied.

## Conclusions

In women with prolonged second stage, the extended duration did not affect the risk of anal incontinence at 1–2 years after delivery. An extended duration of a prolonged second stage should therefore not be an indication to expedite delivery. Vacuum extraction and especially OASIS after a prolonged second stage were associated with anal incontinence. Care should be taken to avoid OASIS and if several risk factors count up, cesarean section is preferable to avoid anal incontinence.

## Supplementary Information

Supplementary Information 1.

Supplementary Information 2.

Supplementary Information 3.

Supplementary Information 4.

## Data Availability

The ethical approval for this study and the informed consent from the stuty participants gave us access to retrieve data to conduct the study, but we were not given permission to share data.

## References

[1] Cheng YW, Hopkins LM, Caughey AB (2004). How long is too long: Does a prolonged second stage of labor in nulliparous women affect maternal and neonatal outcomes?. Am. J. Obstet. Gynecol..

[2] ACOG. Practice bulletin No. 154: Operative vaginal delivery. *Obstet Gynecol* **126**, e56–65 (2015). 10.1097/AOG.0000000000001147 26488523

[3] ACOG. Practice bulletin number 49, December 2003: Dystocia and augmentation of labor. *Obstet Gynecol* **102**, 1445–1454 (2003). 10.1016/j.obstetgynecol.2003.10.011 14662243

[4] WHO. WHO recommendations: Intrapartum care for a positive childbirth experience. (2018). https://www.ncbi.nlm.nih.gov/pubmed/30070803. 30070803

[5] WHO. WHO labour care guide—user’s manual. (2020). https://www.who.int/publications/i/item/9789240017566.

[6] Laughon SK, Berghella V, Reddy UM, Sundaram R, Lu Z, Hoffman MK (2014). Neonatal and maternal outcomes with prolonged second stage of labor. Obstet. Gynecol..

[7] Simic M, Cnattingius S, Petersson G, Sandstrom A, Stephansson O (2017). Duration of second stage of labor and instrumental delivery as risk factors for severe perineal lacerations: Population-based study. BMC Pregnancy Childbirth.

[8] Zhang J, Landy HJ, Ware Branch D, Burkman R, Haberman S, Gregory KD (2010). Contemporary patterns of spontaneous labor with normal neonatal outcomes. Obstet. Gynecol..

[9] Sandström A, Altman M, Cnattingius S, Johansson S, Ahlberg M, Stephansson O (2017). Durations of second stage of labor and pushing, and adverse neonatal outcomes: A population-based cohort study. J. Perinatol..

[10] Allen VM, Baskett TF, O'Connell CM, McKeen D, Allen AC (2009). Maternal and perinatal outcomes with increasing duration of the second stage of labor. Obstet. Gynecol..

[11] Ramm O, Woo VG, Hung YY, Chen HC, Ritterman Weintraub ML (2018). Risk factors for the development of obstetric anal sphincter injuries in modern obstetric practice. Obstet. Gynecol..

[12] Pergialiotis V, Bellos I, Antsaklis A, Papapanagiotou A, Loutradis D, Daskalakis G (2020). Maternal and neonatal outcomes following a prolonged second stage of labor: A meta-analysis of observational studies. Eur. J. Obstet. Gynecol. Reprod. Biol..

[13] Le Ray C, Audibert F, Goffinet F, Fraser W (2009). When to stop pushing: Effects of duration of second-stage expulsion efforts on maternal and neonatal outcomes in nulliparous women with epidural analgesia. Am. J. Obstet. Gynecol..

[14] Looft E, Simic M, Ahlberg M, Snowden JM, Cheng YW, Stephansson O (2017). Duration of second stage of labour at term and pushing time: Risk factors for postpartum haemorrhage. Paediatr. Perinat. Epidemiol..

[15] Stephansson O, Sandstrom A, Petersson G, Wikstrom AK, Cnattingius S (2016). Prolonged second stage of labour, maternal infectious disease, urinary retention and other complications in the early postpartum period. BJOG.

[16] Rouse DJ, Weiner SJ, Bloom SL, Varner MW, Spong CY, Ramin SM (2009). Second-stage labor duration in nulliparous women: Relationship to maternal and perinatal outcomes. Am. J. Obstet. Gynecol..

[17] Myles TD, Santolaya J (2003). Maternal and neonatal outcomes in patients with a prolonged second stage of labor. Obstet. Gynecol..

[18] Infante-Torres N, Molina-Alarcón M, Arias-Arias A, Rodríguez-Almagro J, Hernández-Martínez A (2020). Relationship between prolonged second stage of labor and short-term neonatal morbidity: A systematic review and meta-analysis. Int. J. Environ. Res. Public Health.

[19] Altman M, Sandstrom A, Petersson G, Frisell T, Cnattingius S, Stephansson O (2015). Prolonged second stage of labor is associated with low Apgar score. Eur. J.. Epidemiol..

[20] Garmi G, Peretz H, Braverman M, Berkovich I, Molnar R, Salim R (2016). Risk factors for obstetric anal sphincter injury: To prolong or to vacuum?. Midwifery.

[21] Wall LL (2006). Obstetric vesicovaginal fistula as an international public-health problem. Lancet.

[22] Spong CY, Berghella V, Wenstrom KD, Mercer BM, Saade GR (2012). Preventing the first cesarean delivery: summary of a joint Eunice Kennedy Shriver National Institute of Child Health and Human Development, Society for Maternal-Fetal Medicine, and American College of Obstetricians and Gynecologists Workshop. Obstet. Gynecol..

[23] Caughey AB, Cahill AG, Guise JM, Rouse DJ (2014). Safe prevention of the primary cesarean delivery. Am. J. Obstet. Gynecol..

[24] NICE. Intrapartum care for healthy women and babies (CG190). (2014). https://www.nice.org.uk/guidance/cg190.

[25] Polnaszek BE, Cahill AG (2020). Evidence-based management of the second stage of labor. Semin. Perinatol..

[26] Gimovsky AC, Aizman L, Sparks A, Levine JT (2019). Pushing the limits: Perinatal outcomes beyond prolonged second stage. J. Matern. Fetal Neonatal Med..

[27] Nordenstam J, Altman D, Brismar S, Zetterstrom J (2009). Natural progression of anal incontinence after childbirth. Int. Urogynecol. J. Pelvic Floor Dysfunct..

[28] Bols EM, Hendriks EJ, Berghmans BC, Baeten CG, Nijhuis JG, de Bie RA (2010). A systematic review of etiological factors for postpartum fecal incontinence. Acta Obstet. Gynecol. Scand..

[29] Gimovsky, A. C., Phillips, J. M., Amero, M., Levine, J. & Berghella, V. Prolonged second stage effect on pelvic floor dysfunction: A follow up survey to a randomized controlled trial. *J Matern Fetal Neonatal Med* 1–6 (2021). 10.1080/14767058.2021.1887122 33586572

[30] Johannessen HH, Mørkved S, Stordahl A, Wibe A, Falk RS (2020). Evolution and risk factors of anal incontinence during the first 6 years after first delivery: A prospective cohort study. BJOG.

[31] Graviditetsregistret. Annual report 2019 of the Swedish pregnancy register. (2020). https://www.medscinet.com/GR/uploads/hemsida/dokumentarkiv/Graviditetsregistrets%20Årsrapport%202019_2.0.pdf.

[32] Berg, M., Dykes, A.K., Nordström, L. & Wennerholm, U.B. Rapport 2011:08 Indikation för värkstimulering med oxytocin under aktiv förlossning. (2011). https://www.researchgate.net/publication/256204419_National_medical_guidelines_for_the_use_of_intrapartum_oxytocin_Nationella_medicinska_Indikationer_-_Indikation_for_varkstimulering_med_oxytocin_under_aktiv_forlossning.

[33] Uustal, E. (Swedish perineal tear register, The Swedish national quality register of gynecological surgery).

[34] Jorge JM, Wexner SD (1993). Etiology and management of fecal incontinence. Dis. Colon Rectum.

[35] Wexner SD (2021). Further validation of the Wexner Incontinence Score: A note of appreciation and gratitude. Surgery.

[36] Norderval S, Rydningen MB, Falk RS, Stordahl A, Johannessen HH (2019). Strong agreement between interview-obtained and self-administered Wexner and St. Mark's scores using a single questionnaire. Int. Urogynecol. J..

[37] Jangö H, Langhoff-Roos J, Rosthøj S, Sakse A (2020). Wexner score and quality of life in women with obstetric anal sphincter injury. Int. Urogynecol. J..

[38] von Elm E, Altman DG, Egger M, Pocock SJ, Gøtzsche PC, Vandenbroucke JP (2008). The Strengthening the Reporting of Observational Studies in Epidemiology (STROBE) statement: Guidelines for reporting observational studies. J. Clin. Epidemiol..

[39] Badiou W, Bousquet PJ, Prat-Pradal D, Monroziès X, Mares P, de Tayrac R (2010). Short vs long second stage of labour: Is there a difference in terms of postpartum anal incontinence?. Eur. J. Obstet. Gynecol. Reprod. Biol..

[40] Gimovsky AC, Berghella V (2016). Randomized controlled trial of prolonged second stage: Extending the time limit vs usual guidelines. Am. J. Obstet. Gynecol..

[41] Rogers RG, Leeman LM, Borders N, Qualls C, Fullilove AM, Teaf D (2014). Contribution of the second stage of labour to pelvic floor dysfunction: A prospective cohort comparison of nulliparous women. BJOG.

[42] Brown SJ, Gartland D, Donath S, MacArthur C (2012). Fecal incontinence during the first 12 months postpartum: Complex causal pathways and implications for clinical practice. Obstet. Gynecol..

[43] RCOG. Technical skills tutorial on eLearning and Simulation for Instrumental Delivery (EaSi): Mechanical factors of labour. https://elearning.rcog.org.uk//easi-resource/pelvic-anatomy/mechanical-factors-labour (2022).

[44] Di Mascio D, Saccone G, Bellussi F, Al-Kouatly HB, Brunelli R, Benedetti Panici P (2020). Delayed versus immediate pushing in the second stage of labor in women with neuraxial analgesia: A systematic review and meta-analysis of randomized controlled trials. Am. J. Obstet. Gynecol..

[45] Lemos A, Amorim MM, Dornelas de Andrade A, de Souza AI, Cabral Filho JE, Correia JB (2017). Pushing/bearing down methods for the second stage of labour. Cochrane Database Syst Rev.

[46] Fitzpatrick M, Harkin R, McQuillan K, O'Brien C, O'Connell PR, O'Herlihy C (2002). A randomised clinical trial comparing the effects of delayed versus immediate pushing with epidural analgesia on mode of delivery and faecal continence. BJOG.

[47] Lund NS, Persson LK, Jango H, Gommesen D, Westergaard HB (2016). Episiotomy in vacuum-assisted delivery affects the risk of obstetric anal sphincter injury: A systematic review and meta-analysis. Eur. J. Obstet. Gynecol. Reprod. Biol..

[48] Bergendahl S, Ankarcrona V, Leijonhufvud A, Hesselman S, Karlstrom S, Kopp Kallner H (2019). Lateral episiotomy versus no episiotomy to reduce obstetric anal sphincter injury in vacuum-assisted delivery in nulliparous women: Study protocol on a randomised controlled trial. BMJ Open.

[49] Melendez-Munoz J, Subramanian N, Friedman T, Dietz HP (2020). Is levator trauma an independent risk factor for anal incontinence?. Colorectal Dis..

[50] Valsky DV, Lipschuetz M, Bord A, Eldar I, Messing B, Hochner-Celnikier D (2009). Fetal head circumference and length of second stage of labor are risk factors for levator ani muscle injury, diagnosed by 3-dimensional transperineal ultrasound in primiparous women. Am. J. Obstet. Gynecol..

[51] van Delft K, Thakar R, Sultan AH, Schwertner-Tiepelmann N, Kluivers K (2014). Levator ani muscle avulsion during childbirth: A risk prediction model. BJOG.

[52] Pollack J, Nordenstam J, Brismar S, Lopez A, Altman D, Zetterstrom J (2004). Anal incontinence after vaginal delivery: A five-year prospective cohort study. Obstet. Gynecol..

