# Supplementary material for: Anal incontinence after a prolonged second stage of labor in primiparous women
Source: Sci Rep. 2022 May 5;12:7315. doi: 10.1038/s41598-022-11346-x (PMC9072350; doi:10.1038/s41598-022-11346-x)
Supplement: Supplementary file 1 — Supplementary Information 1. [file 41598_2022_11346_MOESM1_ESM.docx]

**Legend of supplementary tables and figures**

**Supplementary Table S1. Characteristics of women according to mode of delivery**

**Supplementary Table S2. Delivery characteristics and outcome according to mode of delivery**

^a^ No diagnosis, 1^st^ degree or isolated vaginal tear, ^b^ 2^nd^ degree including episiotomy

**Supplementary Figure S1. Directed acyclic graphs**
