# Supplementary material for: Anal incontinence after a prolonged second stage of labor in primiparous women
Source: Sci Rep. 2022 May 5;12:7315. doi: 10.1038/s41598-022-11346-x (PMC9072350; doi:10.1038/s41598-022-11346-x)
Supplement: Supplementary file 2 — Supplementary Information 2. [file 41598_2022_11346_MOESM2_ESM.pptx]

## Slide 1
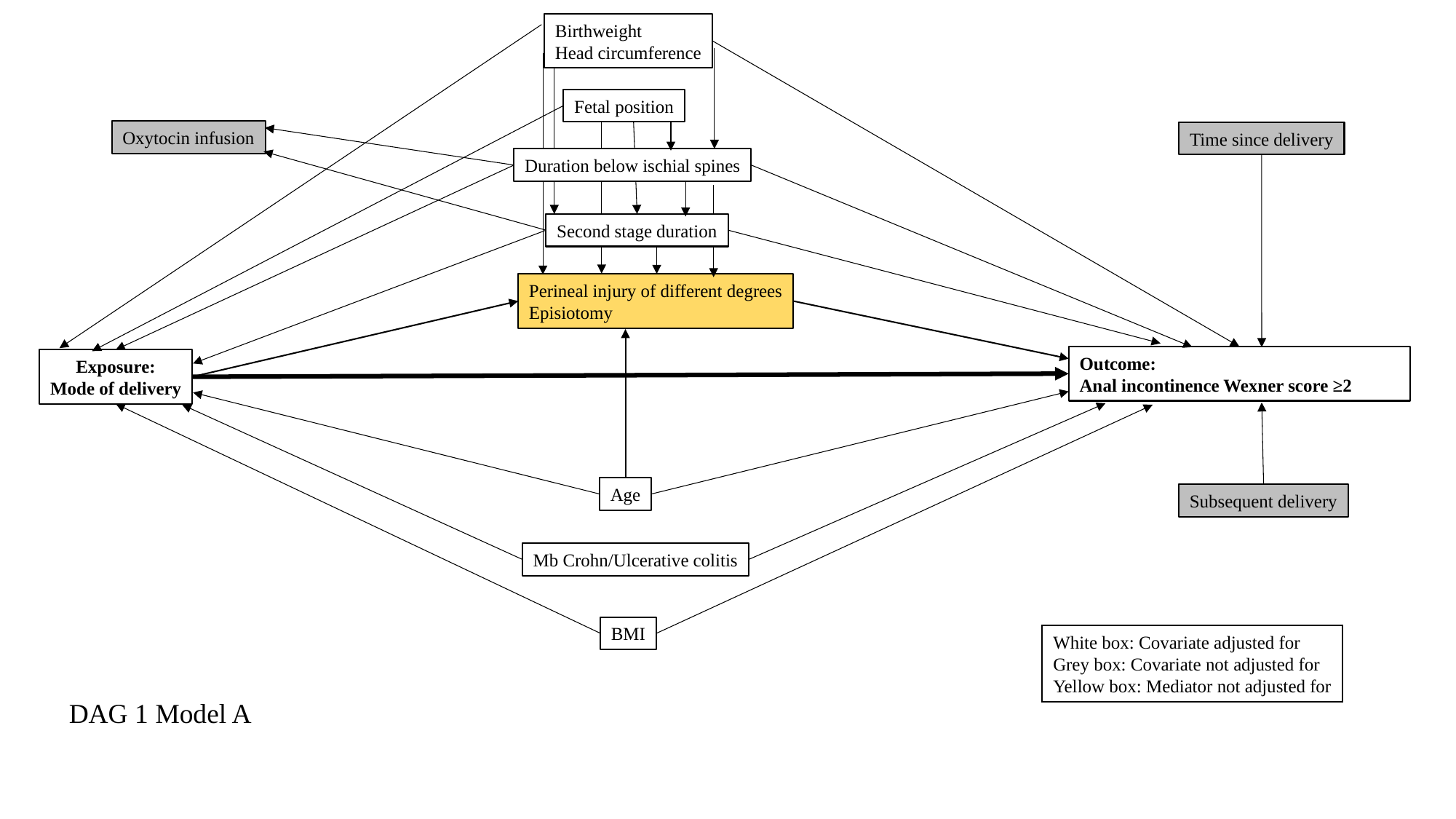

Birthweight
Head circumference
Fetal position
Oxytocin infusion
Time since delivery
Duration below ischial spines
Second stage duration
Perineal injury of different degrees
Episiotomy
Outcome:
Anal incontinence Wexner score ≥2
Exposure:
Mode of delivery
Age
Subsequent delivery
Mb Crohn/Ulcerative colitis
BMI
White box: Covariate adjusted for
Grey box: Covariate not adjusted for
Yellow box: Mediator not adjusted for
DAG 1 Model A

## Slide 2
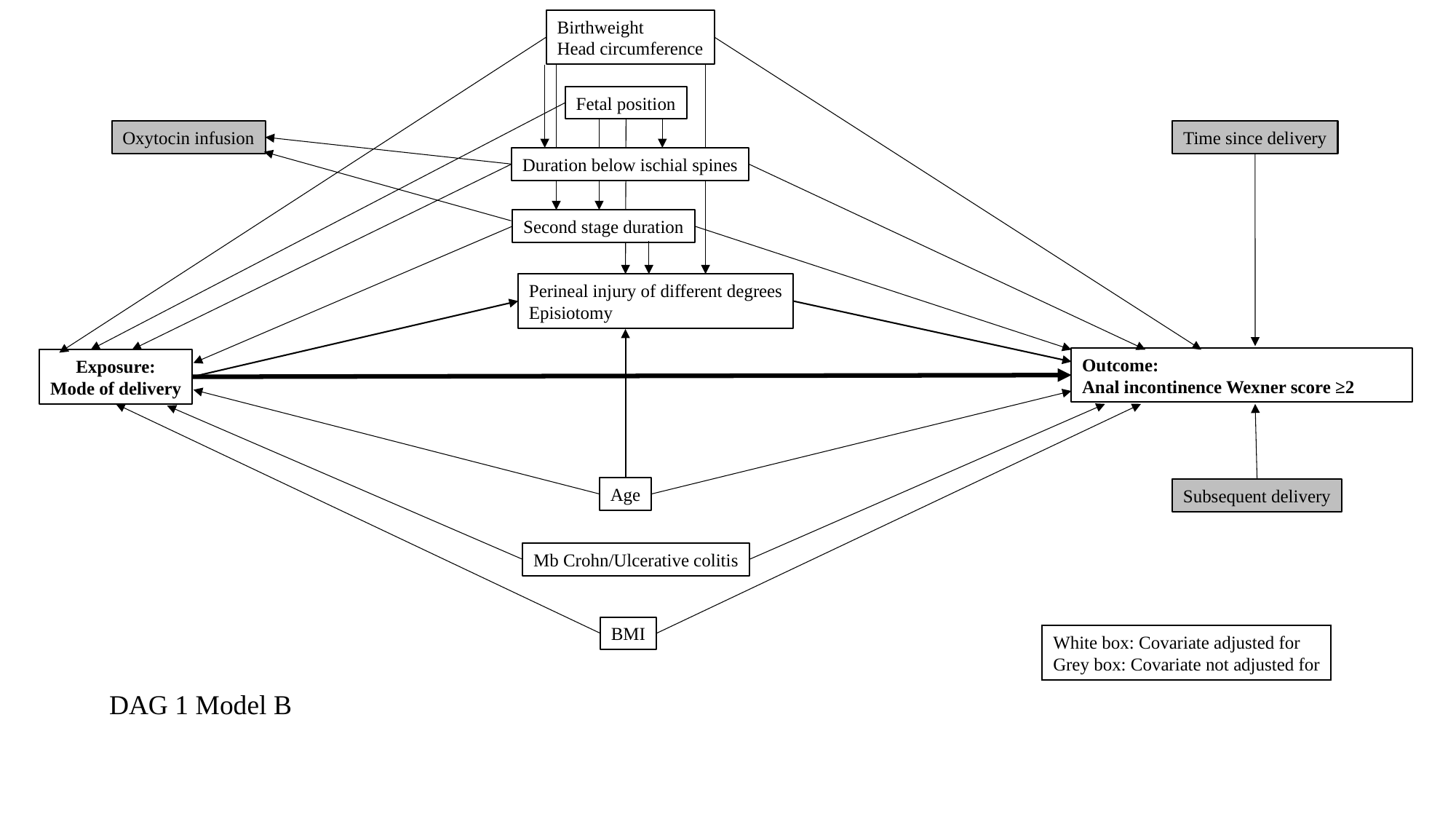

Birthweight
Head circumference
Fetal position
Oxytocin infusion
Time since delivery
Duration below ischial spines
Second stage duration
Perineal injury of different degrees
Episiotomy
Outcome:
Anal incontinence Wexner score ≥2
Exposure:
Mode of delivery
Age
Subsequent delivery
Mb Crohn/Ulcerative colitis
BMI
White box: Covariate adjusted for
Grey box: Covariate not adjusted for
DAG 1 Model B

## Slide 3
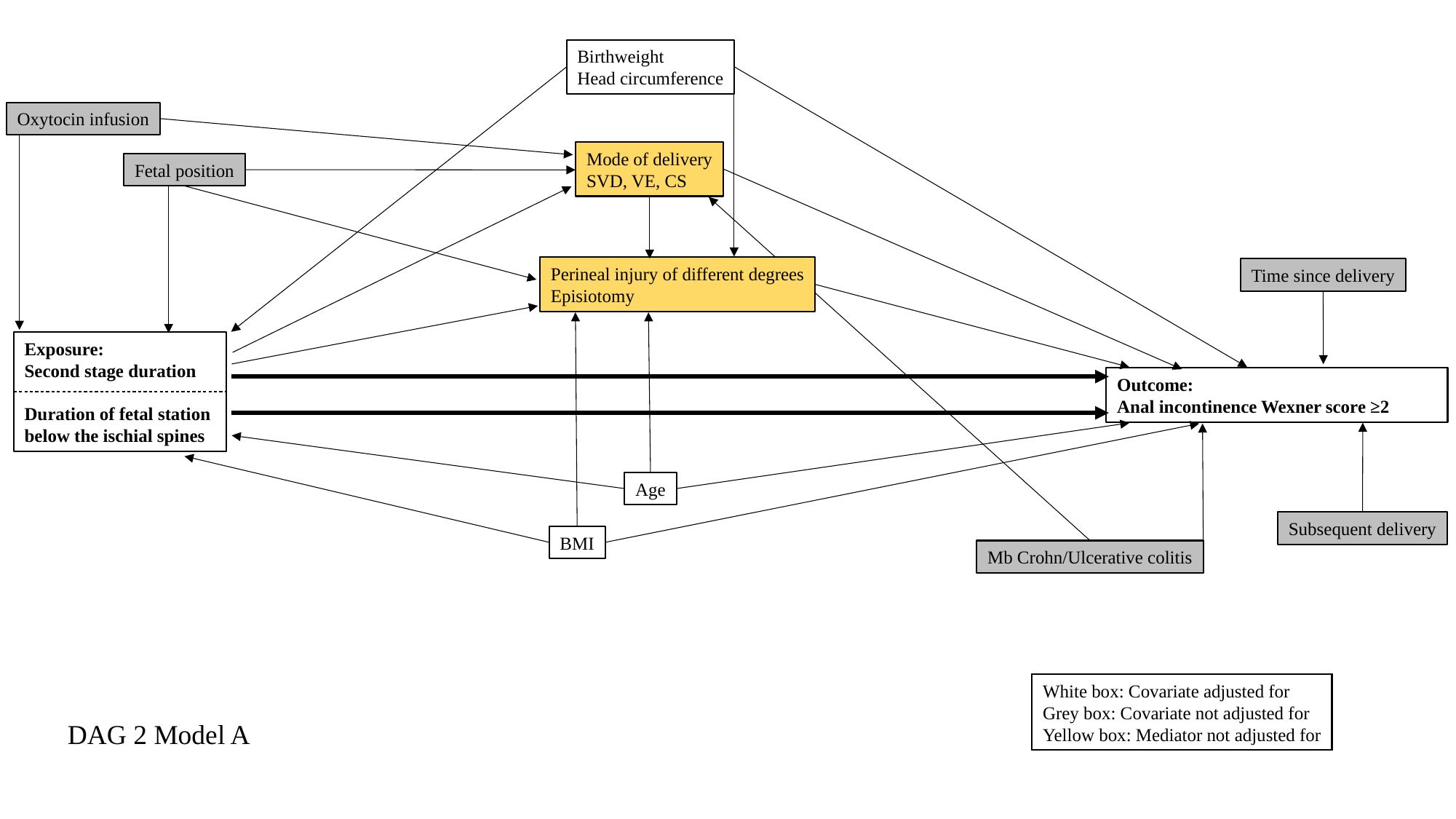

Birthweight
Head circumference
Oxytocin infusion
Mode of delivery
SVD, VE, CS
Fetal position
Perineal injury of different degrees
Episiotomy
Time since delivery
Exposure:
Second stage duration
Duration of fetal station
below the ischial spines
Outcome:
Anal incontinence Wexner score ≥2
Age
Subsequent delivery
BMI
Mb Crohn/Ulcerative colitis
White box: Covariate adjusted for
Grey box: Covariate not adjusted for
Yellow box: Mediator not adjusted for
DAG 2 Model A

## Slide 4
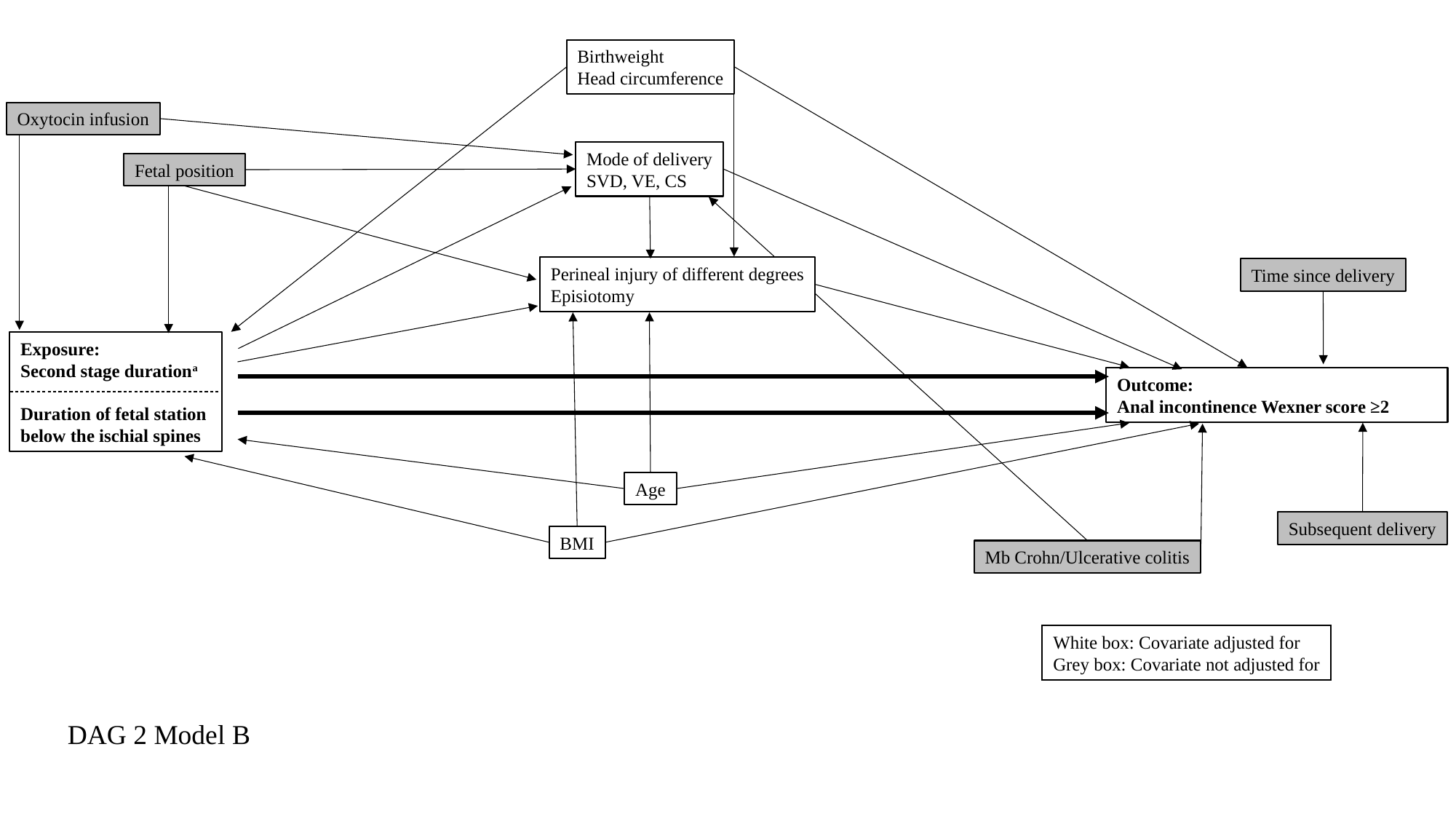

Birthweight
Head circumference
Oxytocin infusion
Mode of delivery
SVD, VE, CS
Fetal position
Perineal injury of different degrees
Episiotomy
Time since delivery
Exposure:
Second stage durationa
Duration of fetal station
below the ischial spines
Outcome:
Anal incontinence Wexner score ≥2
Age
Subsequent delivery
BMI
Mb Crohn/Ulcerative colitis
White box: Covariate adjusted for
Grey box: Covariate not adjusted for
DAG 2 Model B

## Slide 5
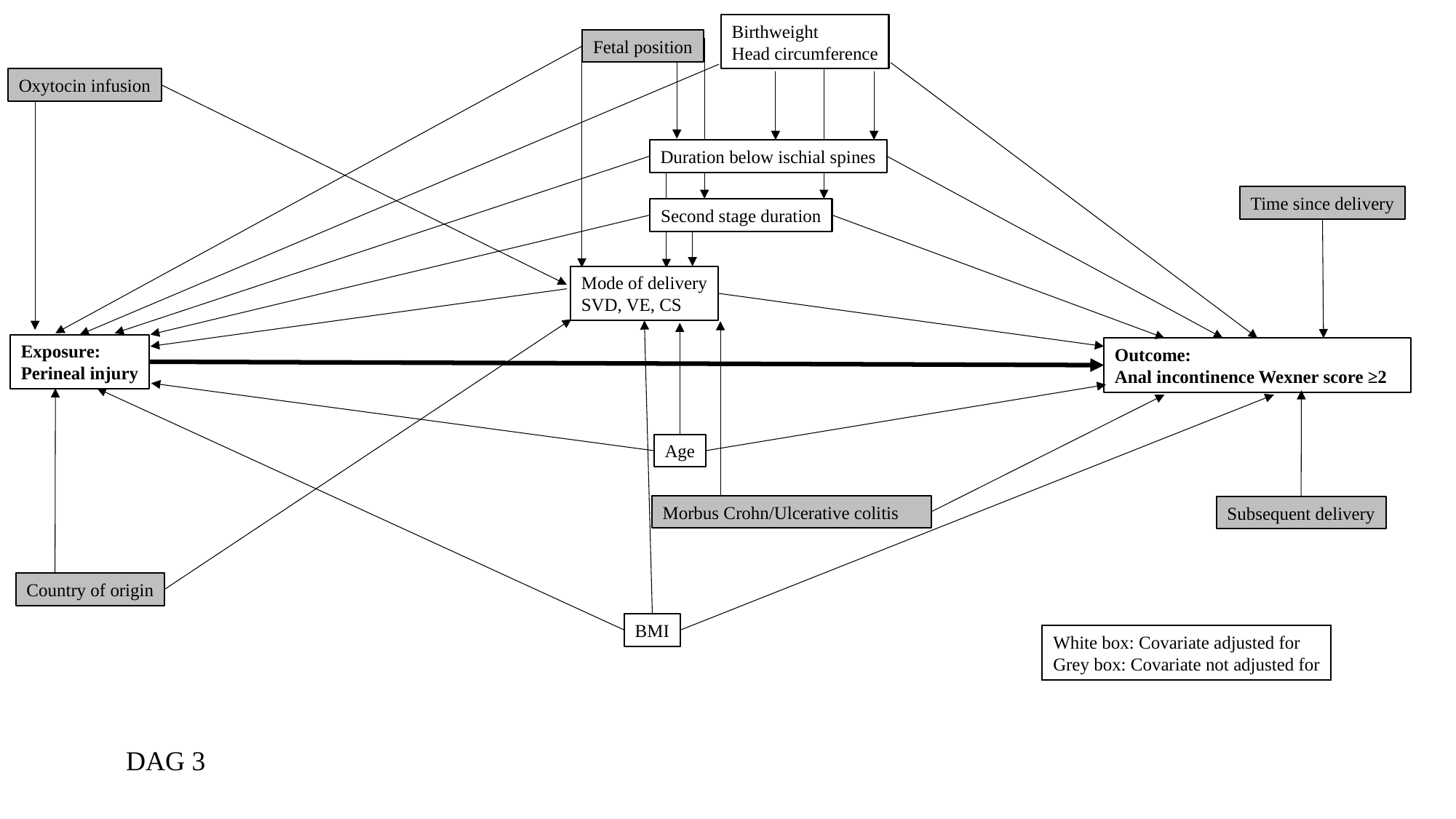

Birthweight
Head circumference
Fetal position
Oxytocin infusion
Duration below ischial spines
Time since delivery
Second stage duration
Mode of delivery
SVD, VE, CS
Exposure:
Perineal injury
Outcome:
Anal incontinence Wexner score ≥2
Age
Morbus Crohn/Ulcerative colitis
Subsequent delivery
Country of origin
BMI
White box: Covariate adjusted for
Grey box: Covariate not adjusted for
DAG 3
