# Supplementary material for: Anal incontinence after a prolonged second stage of labor in primiparous women
Source: Sci Rep. 2022 May 5;12:7315. doi: 10.1038/s41598-022-11346-x (PMC9072350; doi:10.1038/s41598-022-11346-x)
Supplement: Supplementary file 3 — Supplementary Information 3. [file 41598_2022_11346_MOESM3_ESM.docx]

**Table S1. Characteristics of women according to mode of delivery**

|  |  | **Mode of delivery** | | | |
| --- | --- | --- | --- | --- | --- |
|  |  | **Spontaneous delivery**  **n (%)**  923 (70.9%) | **Vacuum extraction**  **n (%)**  280 (21.5%) | **Caesarean section**  **n (%)**  99 (7.6%) | p-value |
| Age | <35 years | 758 (82.1) | 215 (76.8) | 66 (66.7) | <0.001 |
|  | ≥35 years | 165 (17.9) | 65 (23.2) | 33 (33.3) |  |
|  |  |  |  |  |  |
| Height | <155 cm | 10 (1.1) | 8 (2.9) | 3 (3.1) | 0.066 |
|  | ≥155 cm | 894 (98.9) | 271 (97.1) | 5 (96.9) |  |
|  | Missing | 19 (2.1) | 1 (0.4) | 1 (1.0) |  |
|  |  |  |  |  |  |
| BMI | <30 | 828 (94.2) | 259 (95.2) | 83 (86.5) | 0.006 |
|  | ≥30 | 51 (5.8) | 13 (4.8) | 13 (13.5) |  |
|  | Missing | 44 (4.8) | 3 (3.0) | 8 (2.9) |  |
|  |  |  |  |  |  |
| Country of birth, n (%) | Sweden | 885 (95.9) | 262 (93.6) | 89 (89.9) | 0.064 |
|  | Other European country | 18 (2.0) | 9 (3.2) | 6 (6.1) |  |
|  | Outside Europe | 20 (2.2) | 9 (3.2) | 4 (4.0) |  |
|  |  |  |  |  |  |
| Cohabitation, n (%) | Yes | 855 (92.6) | 261 (93.2) | 88 (88.9) | 0.176 |
|  | No | 53 (5.7) | 15 (5.4) | 11 (11.1) |  |
|  | Missing | 15 (1.6) | 4 (1.4) | 0 |  |
|  |  |  |  |  |  |
| Tobacco use, n (%) | Yes | 10 (1.1) | 1 (0.4) | 1 (1.0) | 0.379 |
|  | No | 873 (94.6) | 272 (97.1) | 96 (97.0) |  |
|  | Missing | 40 (4.3) | 7 (2.5) | 2 (2.0) |  |
|  |  |  |  |  |  |
| Diabetes, n (%) | Yes | 4 (0.4) | 1 (0.4) | 3 (3.0) | 0.013 |
|  | No | 887 (96.1) | 274 (97.9) | 94 (94.9) |  |
|  | Missing | 32 (3.5) | 5 (1.8) | 2 (2.0) |  |
|  |  |  |  |  |  |
| Morbus Crohn/Ulcerative colitis, n (%) | Yes | 6 (0.7) | 1 (0.4) | 0 | 0.493 |
|  | No | 885 (95.9) | 274 (97.9) | 97 (98.0) |  |
|  | Missing | 32 (3.5) | 5 (1.8) | 2 (2.0) |  |
|  |  |  |  |  |  |
| Asthma, n (%) | Yes | 60 (6.5) | 23 (8.2) | 9 (9.1) | 0.420 |
|  | No | 831 (90.0) | 252 (90.0) | 88 (88.9) |  |
|  | Missing | 32 (3.5) | 5 (1.8) | 2 (2.0) |  |
|  |  |  |  |  |  |
| Time since delivery (months) | <18 | 86 (9.4) | 24 (8.7) | 12 (12.1) | 0.599 |
|  | ≥18 | 828 (90.6) | 253 (91.3) | 87 (87.9) |  |
|  | Missing | 9 (1.0) | 3 (1.1) | 0 |  |
|  |  |  |  |  |  |
| Subsequent delivery | Yes | 109 (12.4) | 26 (9.7) | 12 (12.8) | 0.472 |
|  | No | 771 (87.6) | 242 (90.3) | 82 (87.2) |  |
|  | Missing | 43 (4.7) | 12 (4.3) | 5 (5.1) |  |
