# Supplementary material for: Anal incontinence after a prolonged second stage of labor in primiparous women
Source: Sci Rep. 2022 May 5;12:7315. doi: 10.1038/s41598-022-11346-x (PMC9072350; doi:10.1038/s41598-022-11346-x)
Supplement: Supplementary file 4 — Supplementary Information 4. [file 41598_2022_11346_MOESM4_ESM.docx]

**Table S2. Delivery characteristics and outcomes according to mode of delivery**

|  |  | **Spontaneous delivery**  **n (%)**  923 (70.9%) | **Vacuum extraction**  **n (%)**  280 (21.5%) | **Caesarean section**  **n (%)**  99 (7.6%) | **p-value** |
| --- | --- | --- | --- | --- | --- |
| Gestational age (weeks) | 37-38 | 123 (13.3) | 38 (13.6) | 12 (12.1) | 0.041 |
|  | 39-40 | 522 (56.6) | 134 (47.9) | 48 (48.5) |  |
|  | 41-42 | 278 (30.1) | 108 (38.6) | 39 (39.4) |  |
|  |  |  |  |  |  |
| Mode of onset | Induction of labor | 195 (21.1) | 86 (30.7) | 42 (42.4) | <0.001 |
|  | Spontaneous onset | 728 (78.9) | 194 (69.3) | 57 (57.6) |  |
|  |  |  |  |  |  |
| Epidural anesthesia | Yes | 734 (79.5) | 239 (85.4) | 86 (86.9) | 0.031 |
|  | No | 189 (20.5) | 41 (14.6) | 13 (13.1) |  |
|  |  |  |  |  |  |
| Duration of second stage (h:mm) | 3:00-3:59 | 545 (59.0) | 98 (35.0) | 16 (16.2) | <0.001 |
|  | 4:00-4:59 | 263 (28.5) | 119 (42.5) | 32 (32.3) |  |
|  | 5:00-5:59 | 91 (9.9) | 45 (16.1) | 30 (30.3) |  |
|  | ≥6:00 | 24 (2.6) | 18 (6.4) | 21 (21.2) |  |
|  |  |  |  |  |  |
| Oxytocin augmentation | Yes | 849 (92.0) | 280 (100.0) | 98 (99.0) | <0.001 |
|  | No | 74 (8.0) | 0 | 1 (1.0) |  |
|  |  |  |  |  |  |
| Duration of fetal station below the ischiadic spines (h:mm) | 0:00-00:59 | 178 (21.2) | 38 (13.6) | 4 (4.0) | <0.001 |
|  | 1:00-1:59 | 200 (21.7) | 38 (13.6) | 5 (5.1) |  |
|  | 2:00-2:59 | 133 (14.4) | 38 (13.6) | 2 (2.0) |  |
|  | 3:00-3:59 | 206 (22.3) | 47 (16.8) | 10 (10.1) |  |
|  | 4:00-4:59 | 72 (7.8) | 50 (17.9) | 11 (11.1) |  |
|  | ≥5:00 | 51 (5.5) | 30 (10.7) | 11 (11.1) |  |
|  | Missing | 83 (9.0) | 39 (13.9) | 56 (56.6) |  |
|  |  |  |  |  |  |
| Birthweight (g) | <4000 | 752 (81.6) | 228 (81.4) | 65 (65.7) | 0.001 |
|  | ≥4000 | 170 (18.4) | 52 (18.6) | 34 (34.3) |  |
|  | Missing | 1 (0.1) | 0 | 0 |  |
|  |  |  |  |  |  |
| Head circumference (cm) | <38 | 883 (96.3) | 258 (93.1) | 89 (91.8) | 0.023 |
|  | ≥38 | 34 (3.7) | 19 (6.9) | 8 (8.2) |  |
|  | Missing | 6 (0.7) | 3 (1.1) | 2 (2.0) |  |
|  |  |  |  |  |  |
| Fetal position | Occiput anterior | 870 (94.3) | 258 (92.1) | 55 (55.6) | <0.001 |
|  | Occiput posterior | 37 (4.0) | 16 (5.7) | 29 (29.3) |  |
|  | Other | 9 (1.0) | 4 (1.4) | 10 (10.1) |  |
|  | Missing | 7 (0.8) | 2 (0.7) | 5 (5.1) |  |
|  |  |  |  |  |  |
| Perineal injury | Degree 0-1^a^ | 361 (39.1) | 58 (20.7) | - | <0.001 |
|  | Degree 2^b^ | 525 (56.9) | 187 (66.8) | - |  |
|  | 2^nd^ Degree only | 491 (53.2) | 169 (60.4) | - |  |
|  | Episiotomy only | 34 (3.7) | 18 (6.4) | - |  |
|  | OASIS | 37 (4.0) | 35 (12.5) | - |  |
|  | 3^rd^ Degree | 36 (3.9) | 33 (11.8) | - |  |
|  | 4^th^ Degree | 1 (0.1) | 2 (0.7) | - |  |
|  |  |  |  |  |  |
| Episiotomy | Yes | 68 (7.4) | 60 (21.4) | - | <0.001 |
|  | No | 855 (92.6) | 220 (78.6) | - |  |

^a^ No diagnosis, 1^st^ degree or isolated vaginal tear, ^b^ 2^nd^ degree including episiotomy
